# Thermal Stability and Magnetic Properties of Polyvinylidene Fluoride/Magnetite Nanocomposites

**DOI:** 10.3390/ma8074553

**Published:** 2015-07-22

**Authors:** Zen-Wei Ouyang, Erh-Chiang Chen, Tzong-Ming Wu

**Affiliations:** Department of Materials Science and Engineering, National Chung Hsing University, 250, Kuo Kuang Road, Taichung 402, Taiwan; E-Mails: redfish20614@hotmail.com (Z.-W.O.); erhchiang.chen@gmail.com (E.-C.C.)

**Keywords:** composite materials, piezoelectric responses, thermal properties, magnetic properties

## Abstract

This work describes the thermal stability and magnetic properties of polyvinylidene fluoride (PVDF)/magnetite nanocomposites fabricated using the solution mixing technique. The image of transmission electron microscopy for PVDF/magnetite nanocomposites reveals that the 13 nm magnetite nanoparticles are well distributed in PVDF matrix. The electroactive β-phase and piezoelectric responses of PVDF/magnetite nanocomposites are increased as the loading of magnetite nanoparticles increases. The piezoelectric responses of PVDF/magnetite films are extensively increased about five times in magnitude with applied strength of electrical field at 35 MV/m. The magnetic properties of PVDF/magnetite nanocomposites exhibit supermagnetism with saturation magnetization in the range of 1.6 × 10^−3^–3.1 × 10^−3^ emu/g, which increases as the amount of magnetite nanoparticles increases. The incorporation of 2 wt % magnetite nanoparticles into the PVDF matrix improves the thermal stability about 25 °C as compared to that of PVDF. The effect of magnetite particles on the isothermal degradation behavior of PVDF is also investigated.

## 1. Introduction

Polyvinylidene fluoride (PVDF) has received considerable interest due to its exceptional ferroelectric and piezoelectric properties [1,2]. Semi-crystalline PVDF contains five different crystalline phases known as α, β, γ, δ, and ε phases, depending on the processing conditions [3]. The polymorphs that happen most commonly are the α and β crystalline phases. The α-phase is obtained directly cooling from the melt or by solvent cast at solvent evaporation temperatures above 120 °C. The α-phase contains the antiparallel trans-gauche bond conformation in the unit cell and thus has no net polarization [4,5,6]. On the other hand, the β-phase, with a parallel packing of all-trans conformation in an orthorhombic unit cell, results in a net nonzero dipole moment. The electroactive β-phase is the most polar phase of PVDF. The β-phase can be achieved by stretching the α-phase at the temperature below 100 °C with a draw ratio between 3 and 5 or by casting from the intensely polar solvent at evaporation temperatures below 70 °C or directly by high-pressure quenching from a melt [7]. Solvent evaporation at higher temperature produces a combination of α- and β-phase, with the portion of β-phase decreasing as the temperature increases. Electric field poling is also used to convert the obtained α-phases into the β-phase [8,9,10]. From above studies, the crystalline structure of PVDF is significantly determined by the processing, thermal, or mechanical treatments that the polymer undergoes.

Another approach has been reported to obtain the electroactive β-phase of PVDF, including the use of BaTiO_3_, TiO_2_, clay, hydrated ionic salt, or ferrite nanoparticles to fabricate the polymer nanocomposites [11,12,13,14,15,16,17,18,19,20,21]. Polymer nanocomposites have relatively high aspect ratio and demonstrate excellent physical properties at lower loading of inorganic fillers [22]. The presence of nanofillers in PVDF leads to significant changes of charge distribution and transportation of the dielectric materials due to the nature of inorganic fillers as well as their interfacial effects. It was also informed that the electroactive β-phase of PVDF is nucleated by the presence of ferrite nanoparticles using surface electrostatic interaction [18]. Incorporation of ferrite nanoparticles with magnetic characteristics into PVDF can display both magnetic and piezoelectric properties for the produced composites [19,20,21]. According to previous report by Goncalves *et al.*, they found the addition of 15 nm Fe_2_O_3_ nanoparticles can significantly enhance the nucleation and the content of β-phase [21]. In our previous research, the incorporation of 6 nm Fe_3_O_4_ nanoparticles into PVDF can improve the mechanical and piezoelectric properties of nanocomposites [23].

In the present work, various concentrations of monodispersed 13 nm magnetite nanoparticles served as nucleation site of β-phase were added into PVDF via solution mixing process to obtain PVDF/magnetite composites with magnetoelectric properties. The thermal and magnetic of the PVDF/magnetite composites were characterized. The piezoelectric properties of samples poled with various applied strengths of electrical field were also measured.

## 2. Experimental

### 2.1. Materials

Polyvinylidene fluoride (Molecular weight = 530,000) was obtained from Aldrich. Iron acetylacetonate, phenyl ether and oleic acid were purchased from Acros and all used without further purification. Other reagents, including tetrahydrofuran (THF), and dimethylformide (DMF), were used as received.

### 2.2. Synthesis of Monodispersed Magnetite Nanoparticles

The monodispersed 13 nm magnetite nanoparticles were prepared using the thermal decomposition of a mixture of iron acetylacetonate, oleic acid, 1,2-hexadecanediol oleylamine, and phenyl ether were added into a three-necked bottle and purged with N_2_ to inhibit the effect of oxygen [24]. The mixture was then heated to reaction temperature at 300 °C and kept at this temperature for the desired time. The mixture was precipitated with ethanol, centrifuged to remove the solvent, and redispersed into hexane.

### 2.3. Fabrication of PVDF/Magnetite Composites

The preparation of PVDF/magnetite nanocomposites was operated using a mixed solvent system (THF/DMF). Various amounts of PVDF and magnetite nanoparticles were individually dissolved or dispersed at 60 °C in a mixed solvent at the THF/DMF ratio of 70/30 [23]. When magnetite nanoparticles and PVDF was dissolved or well dispersed, the two solutions were combined and mixed via sonication until the solutions were uniform. The fabricated PVDF/magnetite nanocomposite was then spread on glass substrate to evaporate the solvent at evaporation temperatures of 60 °C and vacuum dried at 60 °C for 24 h.

### 2.4. Characterization of PVDF/Magnetite Composites

X-ray diffraction (XRD) scans were carried out using a Rigaku D/MAX 2000 diffractometer (BRUKER AXS, Inc., Madison, WI, USA) equipped with Ni-filtered CuKα radiation in the reflection mode. The scan ranges of specimens were collected from 2θ = 5°–40° with the increment of 1°/min. The peak position of the XRD spectra was determined using the Peakfit software package. Transmission electron microscopy (TEM) (JEOL Ltd., Tokyo, Japan) recorded on a Hitachi HF-2000 at 200 kV was used to characterize the interface of PVDF/magnetite composites. The PVDF/magnetite specimens for TEM analysis were obtained using the droplet of PVDF/magnetite composites in solution on the surface of carbon-coated copper grid and then air-dried for 2 h. The zeta potential measurement of magnetite nanoparticles was performed using a Malvern Zetasizer Nano ZS Zeta Potential Analyzer (Malvern Instruments Ltd., Worcestershire, UK). The data shown here represented the mean measurement values from at least five tests.

For piezoelectric measurement, the samples of PVDF and PVDF/magnetite composites were first pressed into thin film under 20 MPa to reduce the porosity of samples. The thickness of PVDF and PVDF/magnetite was about 0.035 mm after press. The piezoelectric measurements were performed using a d_33_ piezoelectric coefficient meter (model YE2730A). In this technique, a normal force was applied by placing a load on a metallic tip oriented perpendicular to the film’s surface and the voltage developed across the sample was measured as a function of the applied stress [25,26]. For electric field poling experiments, the samples were incubated in silicon oil bath using the self-design assembly for the poling treatment. After 1 h of electric field poling at 60 °C with various applied electric fields, the piezoelectric response (*d*_33_) of the poling sample was analyzed. The data shown here represented the mean measurement values from at least three samples. Maximum electric field to obtain the reasonable experimental data was 35 MV/m. By applying the electric field larger than 35 MV/m, several defects, such as pinhole or porosity, will observe on the surface of test samples. This might induce the electric crossover during the measurement. The DC magnetic measurements at room temperature were carried out using a Lakeshore 7400 Series vibrating sample magnetometer (VSM) system (Quantum Design Inc., San Diego, CA, USA). All the magnetization data were normalized to the same weight. The thermal degradation behaviors of PVDF and PVDF/magnetite composites were carried out using Perkin Elmer TG/DTA 6300 thermoanalyzer (PerkinElmer, Waltham, MA, USA). All of the samples were examined from room temperature to 1000 °C under nitrogen atmosphere at a heating rate of 10 °C/min. For isothermal degradation, each sample was heated from 30 °C to 100 °C for 3 min to remove the residual water and then was used a heating rate of 10 °C/min to achieve the predetermined degradation temperatures (*T*_ds_) at 350, 370, 390, 410, and 430 °C for various times.

## 3. Results and Discussion

### 3.1. Characterization and Physical Properties of PVDF/Magnetite Composites

**Figure 1:**
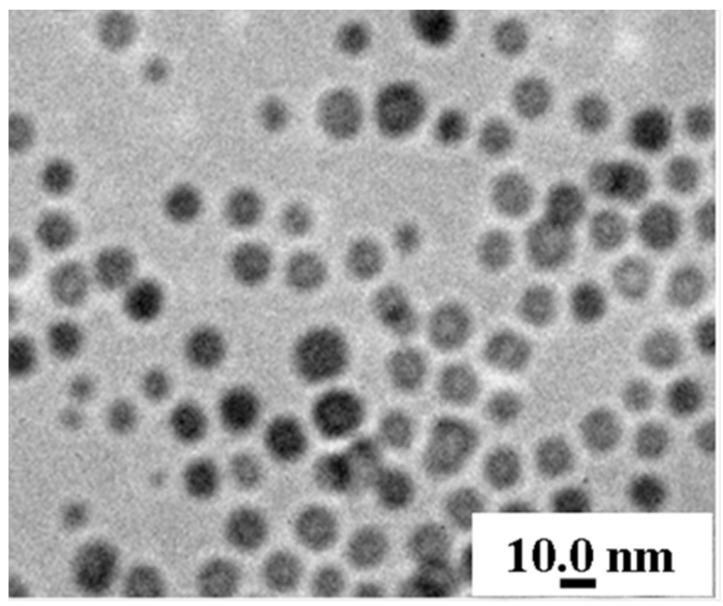
TEM images of monodispersed 13 nm magnetite nanoparticles.

**Figure 2:**
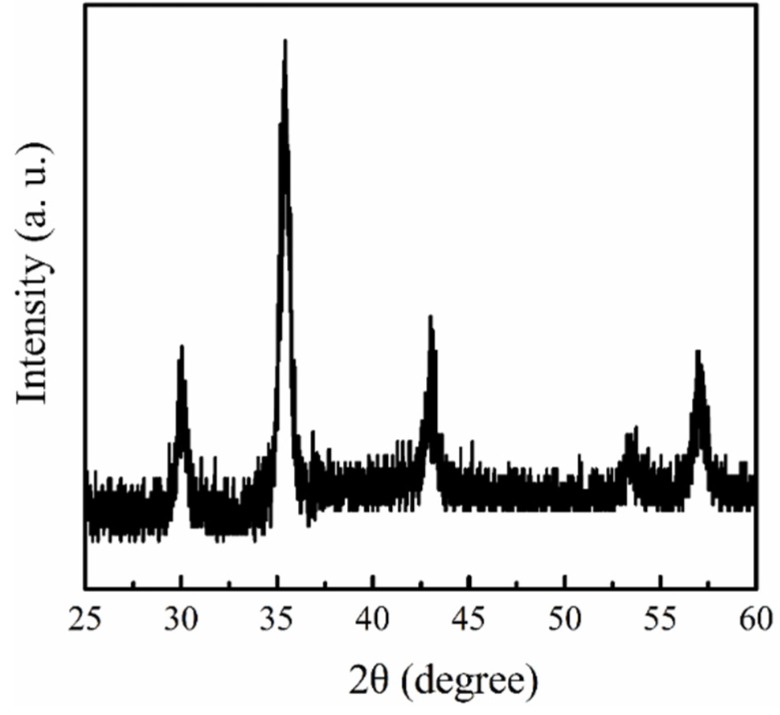
X-ray diffraction pattern of magnetite nanoparticles.

Figure 1 shows the TEM images of 13 nm magnetite synthesized using the thermal decomposition method. From this result, it can be seen that the distribution of particle size is extremely uniform and the average diameter of magnetite particle is about 13 ± 0.9 nm. The X-ray diffraction pattern of magnetite presented in Figure 2 contains six strong diffraction peaks at 2θ = 30.2°, 35.5°, 43.2°, 53.5°, 57.1° and 62.9°, corresponding to (220), (311), (400), (422), (511), and (440) crystalline planes of magnetite phase, respectively [27]. According to the Scherrer’s equation, *t* = λ/βcosθ, an average particle size (*t*) can be estimated from the X-ray wavelength of the Cu Kα radiation (λ), the Bragg angle (θ), and the full width of the peak at half maximum (β) in radians. The particle size of the synthesized magnetite particle using the Scherrer’s equation is 12.8 ± 0.6 nm, which is very close to the data of TEM images. Both TEM and XRD results demonstrate the 13 nm magnetite nanoparticles containing extremely uniform distribution were successfully fabricated using thermal decomposition method.

**Figure 3:**
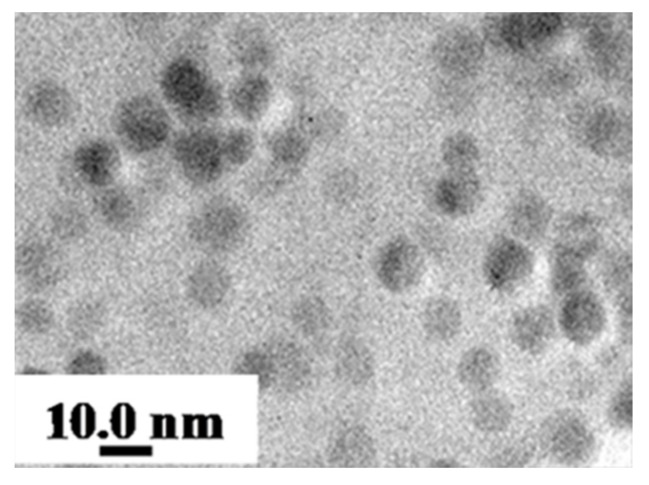
TEM image of the 2 wt % PVDF/magnetite composites.

To reveal the possible interaction between polymer chain of PVDF and magnetite nanoparticles, zeta potential analysis was used to evaluate the electrostatic charge on the surface of prepared magnetite nanoparticles. Because the surface charge of nanoparticles is significantly dependent on the pH of the suspension, the zeta potential of the nanoparticles measured at pH~6 revealed negative surface charge (−12.0 ± 1.1 mV) which is favored to form hydrogen bonding for the PVDF and magnetite nanoparticles. Figure 3 reveals the TEM image of the fabricated PVDF/magnetite nanocomposites. Clearly, a cluster containing several uniformly distributed magnetite nanoparticles is obtained. This result shows that the 13 nm magnetite nanoparticles are well distributed in PVDF matrix.

To investigate the influence of the magnetite nanoparticles on the electroactive β-phase of PVDF, FTIR has been used to calculate the ratio of β-phase for the PVDF/magnetite composites. Particular absorption bands at 764 and 840 cm^−1^ contributed to CF_2_ bending/skeletal bending and CH_2_ rocking have been recognized qualitatively and quantitatively to characterize the α- and β-phase, respectively [28]. Presuming that the infrared absorption follows the Lambert-Beer law for a system including α- and β-phases, the relative fraction of β-phase, *F*(β), can be obtained using Equation (1)

**Figure 4:**
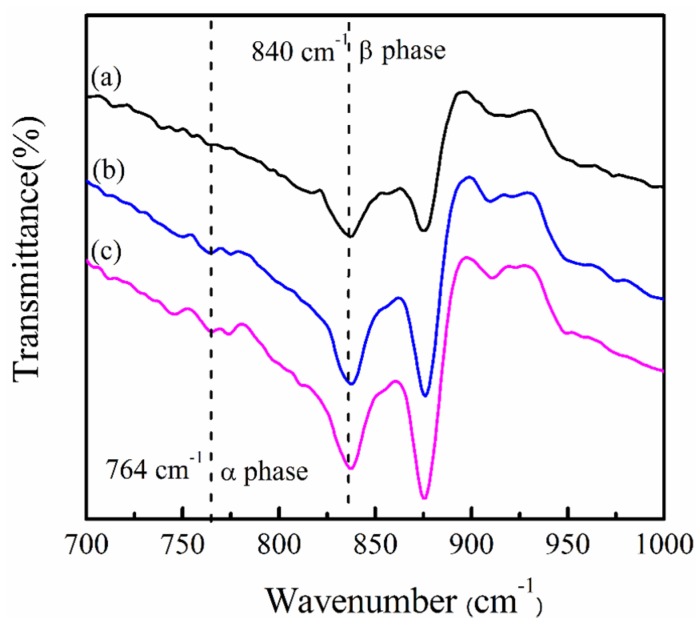
FT-IR spectra of (**a**) PVDF, (**b**) 1 wt % PVDF/magnetite and (**c**) 2 wt % PVDF/magnetite composites.

(1) F(β)=XβXα+Xβ=Aβ(KβKα)Aα+Aβ

where *X*_α_ and *X*_β_ are mass fraction of α and β crystalline phases and *K*_α_ (6.1 × 10^4^ cm^2^/mol) and *K*_β_ (7.7 × 10^4^ cm^2^/mol) are the absorption coefficients at the particular wavenumber, and *A*_α_ and *A*_β_ are the area of absorption bands at 764 and 840 cm^−1^, respectively [5,15]. The FTIR measurements of PVDF/magnetite composites with different weight ratio magnetite particles are shown in Figure 4. For pure PVDF matrix, the FTIR data presented both α and β crystalline phases coexist in the polymer matrix. The relative fraction of the β-phase determined using Equation (1) is 68.8% ± 0.6%, 74.0% ± 0.5%, and 75.3% ± 0.7% for PVDF, 1 wt % PVDF/magnetite and 2 wt% PVDF/magnetite composites, respectively. These results indicated that the PVDF polymer crystallized from the mixed solvent system included α- and β-phase coexist and the amount of β-phase increases with increasing magnetite content. Because of Figure 4, it can conclude that the incorporation of 13 nm magnetite nanoparticles can increase the β-phase fraction which is analogous to previous report using 15 nm Fe_2_O_3_ [18]. These results are attributed to the presence of small particle size of magnetite with negative surface charge. To investigate the effect of the magnetite nanoparticles on the piezoelectricity of specimens, the coefficients of piezoelectric responses (*d*_33_) of PVDF and PVDF/magnetite composites measured using a *d*_33_ piezoelectric coefficient meter were 7.3, 7.6, and 7.8 pC/N, respectively. The obtained piezoelectric coefficients are consistent with previous investigation using a similar preparation method [29]. The value of *d*_33_ was slightly increased with the addition of the magnetite particle. From these results, the incorporation of magnetite nanoparticles into PVDF increased the fraction of β-phase of PVDF in addition to their piezoelectricity.

Electric field poling can be used to convert the obtained α-phases into the β-phase according to previous studies [8,9,10]. To obtain the optimal condition of piezoelectric PVDF/magnetite film, the influence of electric field poling (*E*_p_) on the change of the coefficient of piezoelectric (*d*_33_) was investigated. The piezoelectricity of PVDF and PVDF/magnetite nanocomposites with various strengths of applied electric field were summarized in Table 1. All data suggested that the value of *d*_33_ increased with increasing the strength of electric field. For instance, when electric field poling at 35 MV/m was chose as a point for comparison, the values of *d*_33_ for poling samples are about five times in magnitude higher than the samples without electric field poling. The obtained *d*_33_ values are almost the same order as those reported in the literatures, but the used electric field is much smaller [30,31,32]. The *d*_33_ value of nanocomposites using the same experimental condition of electric field poling was higher than the data of pure PVDF matrix, which might be attributed to the incorporation of magnetite nanoparticles with negative surface charge.

**Table 1:** The coefficient of piezoelectric response (*d*_33_) of PVDF and PVDF/magnetite nanocomposites with various strengths of electric field.

*d*_33_ (pC/N)					
Electrical Field Poling	0 MV/m	14 MV/m	21 MV/m	28 MV/m	35 MV/m
PVDF	7.3 ± 0.1	14.6 ± 0.2	19.2 ± 0.2	31.4 ± 0.4	33.4 ± 0.4
1 wt % PVDF/magnetite	7.6 ± 0.1	22.4 ± 0.2	24.5 ± 0.3	36.7 ± 0.4	37.6 ± 0.4
2 wt % PVDF/magnetite	7.8 ± 0.1	26.4 ± 0.2	28.2 ± 0.3	39.8 ± 0.5	40.6 ± 0.5

**Figure 5:**
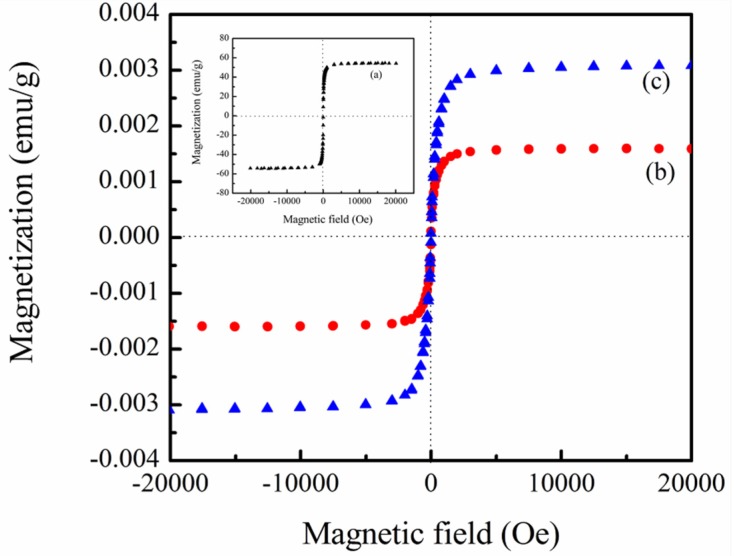
Dependence of applied magnetic field on the saturated magnetization of (**b**) 1 wt % PVDF/magnetite and (**c**) 2 wt % PVDF/magnetite composites (inset curve (**a**) is the dependence of applied magnetic field on the saturated magnetization of 13 nm magnetite particles).

The magnetic properties of PVDF/magnetite composites were determined using VSM system. Characteristic magnetization curves of PVDF/magnetite composites with the applied magnetic field at room temperature are shown in Figure 5. For comparison, the magnetic property of 13 nm magnetiteis also shown in this figure, which points to supermagnetism with saturation magnetization (*Ms*) of 54.2 emu/g. Since the PVDF is not magnetic material, the magnetic properties of the produced composites strongly rely on the contents of magnetite nanoparticles. The magnetic properties of fabricated composites reveal supermagnetism with *Ms* of 1.6 × 10^−3^ and 3.1 × 10^−3^ emu/g for 1 wt % and 2 wt % PVDF/magnetite composites, respectively.

### 3.2. Thermal Stability of PVDF/Magnetite Composites

**Figure 6:**
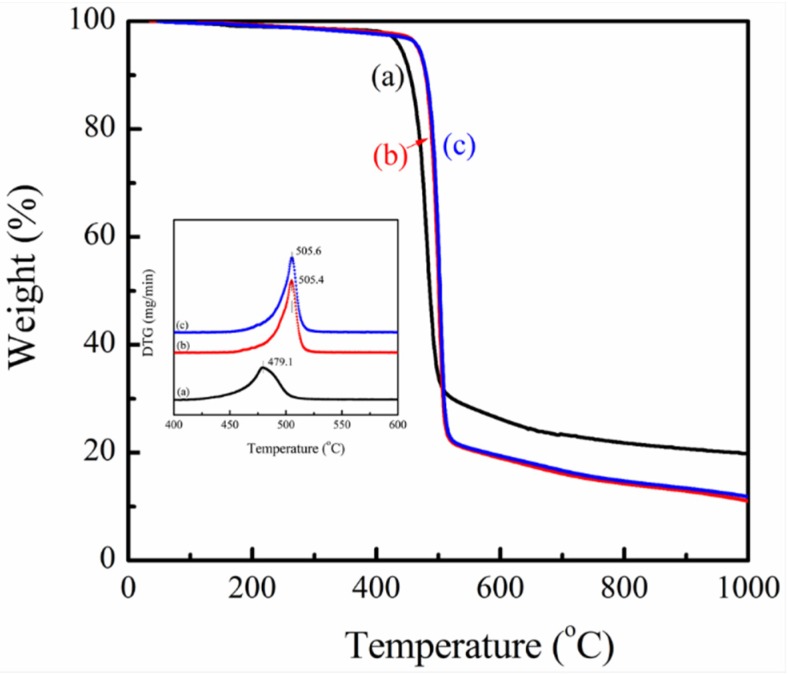
TGA thermograms of (**a**) PVDF, (**b**) 1 wt % PVDF/magnetite and (**c**) 2 wt % PVDF/magnetite composites (inset is the derivative thermogravimetric (DTG) curve of PVDF and PVDF/magnetite composites).

The thermal properties of PVDF/magnetite composites could be performed using TGA analysis. Figure 6 shows the weight loss profiles of PVDF and PVDF/magnetite composites at a heating rate of 10 °C/min. As all experimental data of PVDF and PVDF/magnetite composites have almost the same shape, PVDF/magnetite nanocomposites seem to be more stable if we compare its TGA curve with the curve of pure PVDF matrix in the temperature range of 420–520 °C. The temperature corresponding to maximum degradation rate (*T*_max_) was obtained by the peak position of derivative thermogravimetric curve. Apparently, the *T*_max_ of the PVDF/magnetite nanocomposites is higher than that of pure PVDF matrix. The *T*_max_ of PVDF is 479.1 ± 0.5 °C and extensively increases to 505.4 ± 0.7 and 505.6 ± 0.7 °C for the magnetite loading of 1 and 2 wt %, respectively. Generally, the incorporation of magnetite nanoparticles increased thermal stability about 25 °C as compared to that of PVDF. From these experimental results, it suggests that the incorporation of magnetite nanoparticles in PVDF induced better thermal stability and thus the starting temperature of degradation noticeably moved to higher temperatures. This fact is associated with the inorganic nature of magnetite with uniform distribution, which supplied the superior interference of heat sources, thus improves the thermal stability. Possible degradation behavior will be investigated using isothermal degradation experiments.

**Figure 7:**
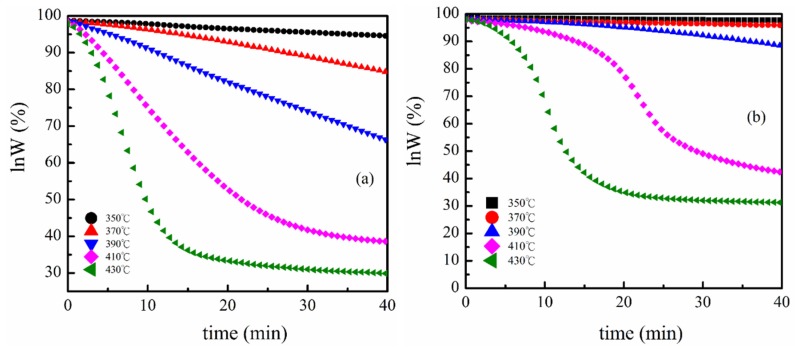
(**a**) Plot of ln*W* against *t* of PVDF at various isothermal temperatures; (**b**) Plot of ln*W* against *t* of 2 wt % PVDF/magnetite composites at various isothermal temperatures.

The isothermal degradation behaviors of PVDF and PVDF/magnetite composites were investigated at predetermined temperatures. The weight loss profiles of PVDF and PVDF/magnetite composites during isothermal heating at numerous temperatures are recorded. The remaining weight decreased with increasing the heating times and the higher isothermal temperatures were believed to produce the superior weight loss. The isothermal degradation kinetics of PVDF and PVDF/magnetite composites can be analyzed using the well known Freeman and Carroll’s method to establish the order and activation energy of the degradation [33]. The typical degradation form is

(2) $$\frac{-\text{d}W}{\text{d}t}=k_{\text{d}}W^{\text{n}}$$

where *W* is the remaining weight, *k*_d_ is the degradation rate constant and *n* is the order of the degradation. If the degradation behavior follows first order decomposition, then the equation (2) may be transformed into

(3) $$\ln W_{0}-\ln W=k_{\text{d}}\cdot t$$

where the *W*_0_ is initial weight. Plots of ln*W* against *t* for PVDF and 2 wt % PVDF/magnetite composites are illustrated in Figure 7. It can be found that a straight line is observed for PVDF isothermal degradation at 350 °C. This result indicates that the degradation at this temperature can be assigned as a first order decomposition with a steady-state rate constant. As the degradation temperatures continually increase to 390 °C, the plots of ln*W versus t* display similar tendency. Nevertheless, the TGA curve for the isothermal degradation at 410 °C has a concave shape with the degradation rate becoming smaller at higher *t*, demonstrating that either the order of reaction is not one, or the rate constant is not a constant, or both. The degradation behavior of 2 wt % PVDF/magnetite composites assigned as first order decomposition was observed using isothermal degradation in the temperature range also from 350 °C to 390 °C.

**Figure 8:**
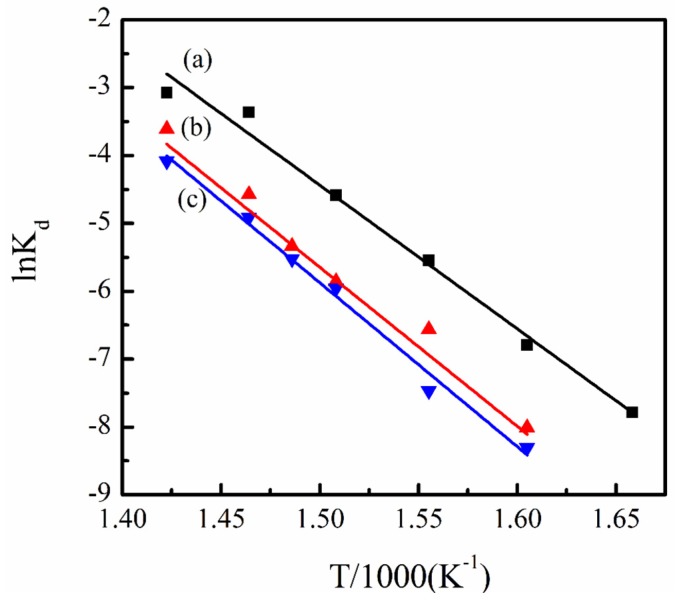
Arrhenius plots of the ln*k*_d_ *versus* 1/*T* of (**a**) PVDF, (**b**) 1 wt % PVDF/magnetite and (**c**) 2 wt % PVDF/magnetite composites.

Using Freeman and Carroll’s model, the kinetic parameters of the degradation were determined assuming a first order decomposition. The degradation rate constant described by Arrhenius equation can be rewritten as follows:

(4) $$\ln k_{\text{d}}=\ln A-\frac{E_{\text{d}}}{RT}$$

where *E*_d_ is the activation energy, *A* is the pre-exponential factor. Figure 8 represents the Arrhenius plots of the ln*k*_d_ against 1/*T* of PVDF and PVDF/magnetite composites. The values of *E*_d_ determined using curve fitting of the experimental data are 175.9, 196.2 and 200.6 kJ/mole for PVDF, 1 wt % PVDF/magnetite and 2 wt % PVDF/magnetite composites, respectively. The *E*_d_ value of PVDF/magnetite composites is significantly changed as compared to that of PVDF. This can be attributed to the introduction of magnetite to PVDF needing more thermal energy for degradation, thus inducing a decrease in the degradation rate and an increase in the residual weight for PVDF/magnetite composites. Nevertheless, the *E*_d_ values of PVDF and PVDF/magnetite composites did not significantly change, suggesting that the addition of small amount magnetite into PVDF matrix does not change the degradation mechanism of PVDF.

## 4. Conclusions

This study describes the thermal stability and magnetic properties of polyvinylidene fluoride (PVDF)/magnetite composites fabricated using solution mixing process. Structural and morphological analysis using transmission electron microscopy showed that the 13 nm magnetite nanoparticles are uniformly distributed in PVDF matrix. The electroactive β-phase and piezoelectric responses of PVDF/magnetite nanocomposites are increased as the loading of magnetite nanoparticles increases. The piezoelectric responses of PVDF/magnetite nanocomposites were extensively increased about five times in magnitude with applied strength of the electrical field at 35 MV/m. The magnetic properties of magnetite coated PVDF nanocomposites exhibit supermagnetism with saturation magnetization in the range of 1.6 × 10^−3^ ~ 3.1 × 10^−3^ emu/g, which increases as the amount of magnetite nanoparticles increases. The temperature corresponding to maximum degradation rate of PVDF/magnetite composites increases with increasing the amounts of magnetite nanoparticles. The incorporation of 2 wt % magnetite nanoparticles into the PVDF matrix enhanced thermal stability by about 25°C as compared to that of PVDF. This is can be ascribed to the incorporation of inorganic iron oxide nanoparticles to PVDF requiring more thermal energy for degradation, thus causing a decrease in the degradation rate and an increase in the residual weight for PVDF/magnetite composites.
