# Correction: Thermal Stability and Magnetic Properties of Polyvinylidene Fluoride/Magnetite Nanocomposites. *Materials* 2015, *8*, 4553–4564

**DOI:** 10.3390/ma8105371

**Published:** 2015-10-22

**Authors:** Zen-Wei Ouyang, Erh-Chiang Chen, Tzong-Ming Wu

**Affiliations:** Department of Materials Science and Engineering, National Chung Hsing University, 250, Kuo Kuang Road, Taichung 402, Taiwan; redfish20614@hotmail.com (Z.-W.O.); erhchiang.chen@gmail.com (E.-C.C.)

In the published manuscript, “Thermal Stability and Magnetic Properties of Polyvinylidene Fluoride/Magnetite Nanocomposites. Materials 2015, 8(7), 4553–4564” [1], we detected that in three places the explanations were slightly incorrect. We apologize for any inconvenience this may have caused.

Page 4556, Section “3.1. Characterization and Physical Properties of PVDF/Magnetite Composites”

Please replace: The X-ray diffraction pattern of magnetite presented in Figure 2 contains six strong diffraction peaks at 2θ = 30.2°, 35.5°, 43.2°, 53.5°, 57.1°and 62.9°, corresponding to (220), (311), (400), (422), (511), and (440) crystalline planes of magnetite phase, respectively [27].

With: The X-ray diffraction pattern of magnetite presented in Figure 2, though affected by a high signal/noise ratio, contains five strong diffraction peaks at 2θ = 30.2, 35.5, 43.2, 53.5 and 57.1, corresponding to (220), (311), (400), (422) and (511) crystalline planes in the magnetite phase [27].

Page 4558, Section “3.1. Characterization and Physical Properties of PVDF/Magnetite Composites”

Please replace: The piezoelectricity of PVDF and PVDF/magnetite nanocomposites with various strengths of applied electric field were summarized in Table 1. All data suggested that the value of *d*_33_ increased with increasing the strength of electric field.

With: The piezoelectricity of PVDF and PVDF/magnetite nanocomposites with various strengths under applied electric field are summarized in Table 1. The standard deviations of the measurements for each sample, as shown in Table 1, are much lower than the resolution of the technique (±1 pC/N). All data suggests that the value of *d*_33_ increased on increasing the strength of the electric field.

Page 4558, Section “3.1. Characterization and Physical Properties of PVDF/Magnetite Composites”

Please replace: The obtained *d*_33_ values are almost the same order as those reported in the literatures, but the used electric field is much smaller [30–32].

With: Though some recent works report higher *d*_33_ values for PVDF under β-phase optimization or other special conditions, the obtained *d*_33_ values are almost of the same order as those currently reported in the literature for PVDF, but the electric field used was much smaller [30–32].
